# Physicochemical, colour, microbiology, sensory and mineral attributes of set-type yoghurt produced from *Gundelia tournefortii* L. and its gum

**DOI:** 10.1007/s13197-024-05987-1

**Published:** 2024-05-03

**Authors:** Dilek Say

**Affiliations:** 1 Vocational School of Pozantı, Cukurova University, 01470 Pozantı, Adana, Turkey; 2 Biotechnology Center, Çukurova University, Adana, Turkey

**Keywords:** *Gundelia tournefortii* L., Yoghurt, Mineral, Colour, Microbiology, External preference map

## Abstract

The present study aimed to investigate the production of yoghurt in which the parts of the *Gundelia tournefortii* L. and its gum were added to milk at levels of 3% (v/v), incubated until the pH value reached 4.7 at 42 °C and then stored at 4 °C for 21 days. The Ca, P, K, Na and Mg contents of the yoghurt sample containing *Gundelia tournefortii* L. proved to be higher than those found in the control sample. Acidity, acetaldehyde, viscosity, a* values of yoghurt samples increased during storage, while the volatile fatty acids, gel firmness, whey separation, water holding capacity, L* and the b* values were decreased. The lactic acid bacteria count on the 21st day was > 6 log CFU/g in all yoghurt samples. The total aerobic mesophilic bacteria decreased except for the yoghurt sample containing *Gundelia tournefortii* L. milk, while the yeast and mold increased. The preference map revealed that 60–80% of consumers were satisfied with the control sample and the *Gundelia tournefortii* L. gum sample. *Gundelia tournefortii* L. milk and *Gundelia tournefortii* L. leaves samples were also found to have acceptable scores. As a result, adding *Gundelia tournefortii* L. into the yoghurt turned out to have a positive impact on its sensory and functional properties and could lead to an innovative approach in the dairy product market.

**Graphical abstract:**

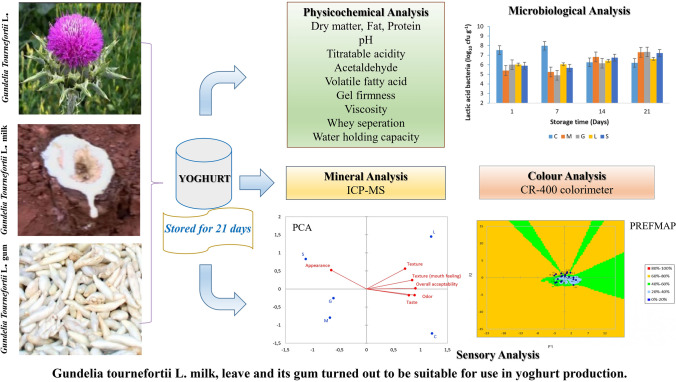

**Supplementary Information:**

The online version contains supplementary material available at 10.1007/s13197-024-05987-1.

## Introduction

Yoghurt is a fermented milk product with high nutritional value produced by lactic acid fermentation using *Lactobacillus bulgaricus* subsp. *delbrueckii* and *Streptococcus thermophiles*. Yoghurt consumption tends to increase all over the world and the most important reason for its acceptance by consumers is its organoleptic properties (Ahmad et al. 2022). Yoghurt also has many health benefits because it contains live microorganisms and the high digestibility and bioavailability of its *protein*, calcium, potassium, riboflavin (B2), pyridoxine (B6), cobalamin (B12) (Bodot et al. 2013).

In recent years, the use of plants and their parts in yoghurt production has improved the product quality and consumers’ interests as well. Yoghurt production from chickpeas, dew droplets and pine cones has been made in Türkiye (Erdoğan et al. 2016). Furthermore, there are several studies reporting the production of yoghurt by using chickpeas and their extract (Bakr 2013; Erdoğan et al. 2016; Abd Rabo et al. 2019; Aguilar-Raymundo and Velez-Ruiz 2019; Güzeler et al. 2019). In this context, the use of fruits, vegetables and medicinal plants during fermentation influence on yoghurt properties such as total phenolic content, antioxidant activity, the viability of lactic acid bacteria and sensory properties (Bakr 2013).

*Gundelia tournefortii* L. from the Asteraceae family is a thistle-like, perennial herb with milky latex that reaches a height of 40–50 cm (Khanzadeh et al. 2012). This medicinal plant is found in mountainous areas of countries such as Azerbaijan, Cyprus, Jordan, Egypt, Israel, Turkiye, Iran, and Turkmenistan (Cakmakcı and Dagdemir 2013). The part of this plant, including stems, flowers, buds, leaves and seeds are consumed as food. Moreover, the chewable gum (*Gundelia tournefortii* L. gum) is obtained by solidification of milk flowing as a result of cutting the root of this plant. It has been used as a remedy for toothache, cramp, indigestion and migraine (Palabıyık et al. 2018). The *Gundelia tournefortii* L. grown in Turkiye are known to have higher protein (12.6%), crude fibre (27.2%), crude oil (16.2%), and Ca, P, K, Na, Mg, Zn, Fe (Cakmakcı and Dagdemir 2013). A famous restaurant in Istanbul, Türkiye, for example, produces yoghurt using the milk of *Gundelia tournefortii* L. and its customers are said to prefer it because of its acidic and aromatic flavour. Ebrahimi and Sani (2015) demonstrated that the addition of different proportions of *Gundelia tournefortii* L. puree to the yoghurt significantly improved its quality. Cakmakcı and Dagdemir (2013) reported that *Gundelia tournefortii* L. leaves and milk can also be used as stabilizers in ice cream production. Khoshvaghtia and Javaheri (2021) found that the yoghurt samples with *Gundelia tournefortii* L. extract had an effect similar to those of probiotic yoghurt samples with *Lb. acidophilus* and *B. bifidum* in the case of hypercholesterolemia induced by the consumption of high-fat diets.

On the other hand, there are very few studies concerning the inclusion of *Gundelia tournefortii* L. in dairy products. Using *Gundelia tournefortii* L. milk and its gum for yoghurt production has not yet been studied either. The objective of this study was to study the outcome of including *Gundelia tournefortii* L. in yoghurt production and to determine its physical, chemical, colour, microbiological and sensory properties during 21 days of storage. Moreover, the mineral content of yoghurt samples was investigated only at first the day of storage. The development of alternative dairy products by adding *Gundelia tournefortii* L. with high nutritional value and health benefits will lead to contribution in the fermented dairy industry.

## Materials and methods

### Materials

*Gundelia tournefortii* L. and its milk were collected from the mountainous areas in Pozantı, Adana, Türkiye between June and July. The underground stem of a *Gundelia tournefortii* L. was cut off with a knife. The plant’s milk was taken into sterile containers and used for yoghurt production. *Gundelia tournefortii* L. leaves and stems were washed, crushed and then stored at 4 ± 1 °C before use. *Gundelia tournefortii* L. gum was provided from a local market in Malatya. The cow’s milk was supplied from research and application farm, Çukurova University (Adana, Türkiye). The skim milk powder was purchased from Enka Co. (Konya, Türkiye). The lyophilized yoghurt starter culture (*Lactobacillus bulgaricus* subsp. *delbrueckii* and *Streptococcus thermophilus,* YC350*)* were provided from Chr. Hansen Co. (Hørsholm, Denmark).

### Yoghurt production

Skim milk powder was incorporated at a rate of 3% (w/v) at 40 °C, pasteurisation was applied to the milk at 90 °C for 5 min and then cooled down to 45 °C. The milk was divided into five equal lots (4 L). *Gundelia tournefortii* L. milk, leave, stem and gum were activated in milk and then used. The first lot was served as a control sample (C) and inoculated with 3% (v/v) yoghurt culture. *Gundelia tournefortii* L. milk (M), *Gundelia tournefortii* L. gum (G), *Gundelia tournefortii* L. leaves (L), and *Gundelia tournefortii* L. stem (S) were added to the yoghurt lots at a rate of 3% (v/v) and thoroughly stirred. All milk mixtures were packed into polyethylene cups (80 mL) and incubated until the pH value reached 4.7 at 42 °C. After incubation, set-type yoghurt samples were stored at 4 °C for 21 days. Three replicate trials were carried out in that yoghurt production.

### Physicochemical analysis

Dry matter was determined by drying the sample in an oven at 100 ± 5 °C until constant mass. Total nitrogen (TN) was determined by the micro Kjeldahl method and the protein content was obtained by multiplying the percentage of TN by 6.38 (AOAC 2016). Fat was revealed by Gerber method (Mistry and Hassan 1992). After dissolved of the protein with sulphuric acid (10 mL, density 1.825 g/L). The addition of a small quantity of amyl alcohol (1 mL, density 0.815 g/L) was aided the separation. The fat content was read directly on butyrometer scale. pH analyses were performed, using the WTW 3110 pH meter (Wielheim, Germany). Titratable acidity was expressed as lactic acid % by titration of 10 mL sample using phenolphthalein indicator (0.1%) and 0.1 N NaOH solution by alkali titration method (IDF 1991). Acetaldehyde was ascertained according to the method specified by Lees and Jago (1969) in conformity with iodometric. The mixture of yoghurt sample (10 g) and pure water (30 mL) were prepared for Kjeldahl process and about 10 ml of distillate was collected. NaHSO_3_ (1 mL) was added to bind acetaldehyde in the distillate and the pH of the mixture was adjusted to 9 with 0.01 N NaOH and kept in a dark place for 15 min. 1% starch solution (1 mL) was added as an indicator and titrated with 0.1 N iodine solution until it turned purple, then NaHSO_3_ (1 g) was added and titrated again with 0.005 N iodine solution until it turned purple. Volatile fatty acids were analysed by Kosikowski (1978). The yoghurt (1 mL) to be analysed was weighed into a digestion flask and added MgSO_4_.7H_2_O (3.5 mL), H_2_SO_4_ (5 mL) and pure water (30 mL). The solution in the digestion flask was then made alkaline by addition of sodium hydroxide. The content was then determined by titration with standard 0.1 N NaOH solution, using phenolphthalein indicator (0.1%) to determine the end-point of the reaction. Gel firmness was measured using a PNR 6 Sur Berlin penetrometer (Berlin, Germany) with a 15 g of conical (45°) probe at 5 s. Viscosity was found, using a DV-II + Pro Brookfield viscometer (MA, USA) with a spindle No. S64 at 100 rpm at 4 °C and the results were given at 15 s and expressed in centipoises. Three repetitions were performed for each yoghurt sample (Shihata and Shah 2002). Whey separation was calculated by the amount of whey drained off from 25 g yoghurt at 4 ± 1 °C for 120 min (Tamime et al. 1996). Water holding capacity (WHC) was carried out using a modified procedure from Remeuf et al. (2003) 5 g of yoghurt sample (Y) was centrifuged for 30 min at 4500 rpm and 10 °C and the pellet (P) was weighed. The WHC was calculated with the formula:

$$WHC \, \left( \% \right) \, = \, \left( {Y - P} \right) \, / \, Y \, \times 100$$

### Mineral analysis

Firstly, yoghurt samples were digested in a microwave oven (Berghof M2S-2, Berghof Products Instruments GmbH, Eningen, Germany) and then macro and micro mineral elements were identified with ICP-MS (Perkin-Elmer, Nexion 2000P, Shelton, CT, USA) (Khan et al. 2014).

### Colour analysis

The colour (L*, a*, b*) of yoghurt samples were performed using a chroma meter (model CR-400, Minolta Camera Co. Ltd, Osaka, Japan). Before analysis, the colorimeter was calibrated with a blank and the results were given as color averages of three different readings.

### Enumeration of microorganisms

Plate count agar (PCA) (Merck, Darmstadt, Germany) was used for enumeration of total aerobic mesophilic bacteria under aerobic incubation at 30 °C for 48 h. Man, Rogosa and Sharpe Agar (MRS) (Merck, Darmstadt, Germany) were used at pH 5.8 for lactic acid bacteria with anaerobic incubation. The plates for lactic acid bacteria counting were kept at 30 °C for 3 days in anaerobic jars containing oxygen-removing gas packages (Anaerocult; Anaerocult^®^ A, Merck). Yeast and mold counts were performed in Potato Dextrose Agar (PDA) (Merck, Darmstadt, Germany). The incubation of yeast and mold was conducted at 25 °C for 72 h and 25 °C for 7 d, respectively (Harrigan 1998).

### Sensory analysis

A quantitative descriptive sensory attribute analysis was performed during the 21 days of storage at 4 °C by a panel of seven experts. The panellists were served all yoghurt samples in plastic cups which had been coded with random two-digit numbers. Additionally, they were provided with bread and water so that they could clean their palate between yoghurt samples. The panallists answered a questionnaire for yoghurt samples. They then scored the samples for the appearance, texture (as perceived by spoon and in the mouth), odour, taste and overall acceptability (scale 1–5) (TSI 2006).

### Statistical analysis

SPSS 22 statistical software was used to perform one-way ANOVA. The differences between the mean rates were determined using the Duncan’s multiple comparison test (*p* < 0.05). All experiments were performed in triplicate and values were presented as a mean ± standard deviation. Furthermore, the Principal Component Analysis (PCA) was conducted and the External Preference Map (PREPMAP) created using Microsoft Excel with XLSTAT (essential data analysis tools for Excel, 2023 version, Addinsoft, USA).

## Results and discussion

### Composition of milk and yoghurt

In Table S1 (supplementary material) results showed that pH, titratable acidity, dry matter, fat, protein contents of raw cow milk used in yoghurt production were 6.78, 0.15% l.a., 11.09%, 2.93%, 4.33%, respectively. No statistical significance was found between the dry matter (between 12.95 and 13.38%), fat (between 2.96 and 3.1%) and protein (between 4.11 and 5.11%) values of the control yoghurt sample and those samples containing *Gundelia tournefortii* L. (*p* > 0.05).

### Mineral profile of the yoghurt samples

Conducting a mineral analysis is necessary for the quality and safety of milk and dairy products. Table 1 shown the results of calcium, phosphorus, potassium, sodium, magnesium, zinc, chrome, manganese, iron, and copper elements of yoghurt samples at the first day of storage. *Gundelia tournefortii* L. is known to be an important mineral source, and this study has revealed that the macro element (Ca, P, K, Na and Mg) contents of the yoghurt sample containing *Gundelia tournefortii* L. yielded higher values when compared to control sample. In a manner similar to the results of this study, Dhawi et al. (2020) reported that the Ca, P, K, Mg, Zn and Fe of yoghurt samples containing fenugreek and moringa seeds and flour was higher. The highest Ca, P, K, Na and Mg minerals were found in the yoghurt sample (M) containing *Gundelia tournefortii* L. milk. In the yoghurt sample (S) containing *Gundelia tournefortii* L. stem, all of these elements were detected at the lowest level. The yoghurt sample (L) containing *Gundelia tournefortii* L. leaves was found to be rich in Ca and P, following the yoghurt sample M. When evaluated in terms of K, Na and Mg minerals, the second yoghurt sample containing these elements in high amounts was the yoghurt sample (G) containing *Gundelia tournefortii* L. gum. Considering these values on the whole, it appeared that the yoghurt samples, namely C, M, G, L and S, which were consumed 100 g per day proved to have met the recommended daily allowance (RDA) in terms of Ca, P and K minerals. Moreover, 100 g of samples M and G can be considerable sources of Mg, providing 59.20% and 57.87% of the RDA. When compared to the control sample, the amount of Zn in the yoghurt samples was found to be higher in the samples G, L and S, while it was found to be lower in the sample M. The highest Zn content was found in the sample G, and the lowest in the sample M. The amounts of Cr, Mn, Fe and Cu in all yoghurt samples seemed to be below < 1. In an assumed yoghurt intake of 100 g/day, the sample G appears to supply the 186% of the RDA for Zn. A statistically significant difference was found between the yoghurt samples in terms of the amounts of Ca, P, K, Na, Mg, and Zn (*p* < 0.01). El-Nawasany (2019) reported that Ca, Mg, Fe and Zn content in the yoghurt sample increased as the proportion of the plant increased in stirred yoghurt samples containing Vitex agnus-*castus*. The results reported by the aforementioned researcher for the Ca and Zn contents were lower than our findings, though higher than those found for the Mg content.

**Table 1:** Macro and micro mineral content of yoghurt samples^*^

Yoghurts	Ca (mg/kg)	P (mg/kg)	K (mg/kg)	Na (mg/kg)	Mg (mg/kg)	Zn (mg/kg)	Cr (µg/kg)	Mn (µg/kg)	Fe (µg/kg)	Cu (µg/kg)
C	1526 ± 43^c^	1406 ± 43^c^	2188 ± 53^d^	743 ± 22^d^	177 ± 5^d^	3.76 ± 0.2^c^	< 1	< 1	< 1	< 1
M	1923 ± 62^a^	1612 ± 46^a^	2762 ± 87^a^	936 ± 28^a^	222 ± 6^a^	1.82 ± 0.3^d^	< 1	< 1	< 1	< 1
G	1787 ± 27^b^	1561 ± 14^a^	2706 ± 26^ab^	913 ± 14^ab^	217 ± 3^a^	18.60 ± 0.1^a^	< 1	< 1	< 1	< 1
L	1817 ± 58^b^	1576 ± 43^a^	2631 ± 68^b^	888 ± 24^b^	207 ± 6^b^	4.64 ± 0.3^b^	< 1	< 1	< 1	< 1
S	1749 ± 31^b^	1490 ± 28^b^	2392 ± 46^c^	810 ± 15^c^	192 ± 5^c^	4.85 ± 0.2^b^	< 1	< 1	< 1	< 1
RDA (mg/day)	800	700	2000		375	10	40	2	14	1

C: control yoghurt M: yoghurt containing *Gundelia tournefortii* L. milk, G: yoghurt containing *Gundelia tournefortii* L. gum, L: yoghurt containing *Gundelia tournefortii* L. leave, S: yoghurt containing *Gundelia tournefortii* L. stem, RDA: recommended daily allowance based on EU Regulations, 2011

^*^Presented values are the means (± SD) of three replicate trials

^a,^^b,c,d^Means that, in the same column, different letters were significantly different at *p* < 0.01

### pH, titratable acidity, acetaldehyde and volatile fatty acid of the yoghurt samples

Table 2 presents the pH, titratable acidity, acetaldehyde and volatile fatty acid of yoghurt samples during storage. Lactose metabolism causes the formation of lactic acid and aromatic compounds, especially acetaldehyde, during the fermentation of yoghurt (Ahmad et al. 2021). Initial pH values in the control yoghurt sample as well as the other yoghurt samples M, G, L and S were 4.49, 4.41, 4.42, 4.55, and 4.51, respectively. These values were 4.33, 4.33, 4.29, 4.50 and 4.46 at the end of the 21st day. There was no statistical difference between the pH values of the control sample and the other yoghurt samples (*p* > 0.05). Güzeler et al. (2019) reported the pH value of a yoghurt sample with chickpeas to be 4.03, a value which was lowest of all yoghurt samples in the study. The highest pH value in yoghurt samples was seen on the first day of storage, though it decreased during storage; however, such a decline was not statistically significant (*p* > 0.05). This decrease in the pH value during storage was reported in the studies by Bakr (2013) and Aguilar-Raymundo and Velez-Ruiz (2019) in yoghurt samples with added chickpea extract, as well as by Ebrahimi et al. (2015), who had added *Gundelia tournefortii* in their yoghurt samples. Titratable acidity values in the range from 0.91 to 1.28% in control and other yoghurt samples. Titratable acidity values of L and S samples were found to be lower when compared to other samples. Such a difference was not statistically significant except for the first day of storage (*p* > 0.05). Further-more, the titratable acidity values of yoghurt samples increased during storage until the 21st day, yet no statistical difference was observed between samples (*p* > 0.05). In the same context, Ebrahimi et al. (2015) reported that the titratable acidity values of the yoghurt samples increased during storage when added *Gundelia tournefortii*. The lactic acid produced as a consequence of the metabolic activity of lactic acid bacteria causes the increase of acidity and the reduction of the pH during cold storage.

**Table 2:** pH, titratable acidity, acetaldehyde and volatile fatty acid of yoghurt samples during storage^*^

	Storage days			
	1	7	14	21
pH				
C	4.49 ± 0.1^Aa^	4.35 ± 0.2^Aa^	4.33 ± 0.1^Aa^	4.33 ± 0.1^Aa^
M	4.41 ± 0.1^Aa^	4.35 ± 0.2^Aa^	4.28 ± 0.2^Aa^	4.33 ± 0.1^Aa^
G	4.42 ± 0.^Aa^	4.36 ± 0.2^Aa^	4.27 ± 0.1^Aa^	4.29 ± 0.1^Aa^
L	4.55 ± 0.1^Aa^	4.49 ± 0.1^Aa^	4.48 ± 0.1^Aa^	4.50 ± 0.1^Aa^
S	4.51 ± 0.1^Aa^	4.45 ± 0.1^Aa^	4.43 ± 0.1^Aa^	4.46 ± 0.1^Aa^
Titratable acidity (LA%)				
C	1.08 ± 0^Aa^	1.14 ± 0.2^Aa^	1.12 ± 0.1^Aa^	1.10 ± 0.1^Aa^
M	1.08 ± 0^Aa^	1.19 ± 0.3^Aa^	1.20 ± 0.2^Aa^	1.15 ± 0.3^Aa^
G	1.09 ± 0^Aa^	1.23 ± 0.3^Aa^	1.20 ± 0.2^Aa^	1.28 ± 0.2^Aa^
L	0.91 ± 0.1^Ba^	0.98 ± 0.2^Aa^	0.96 ± 0.1^Aa^	0.93 ± 0.1^Aa^
S	0.94 ± 0^Ba^	1.00 ± 0.1^Aa^	1.01 ± 0.1^Aa^	1.00 ± 0.1^Aa^
Acetaldehyde (ppm)				
C	4.55 ± 0.2^Aa^	3.50 ± 1.3^Ab^	3.56 ± 0.6^Ab^	2.90 ± 0.2^Ac^
M	3.08 ± 0.2^Bc^	4.62 ± 1.6^Aa^	3.85 ± 1.5^Ab^	4.93 ± 3.6^Aa^
G	2.88 ± 0.9^Bc^	3.56 ± 2^Ab^	2.92 ± 0.6^Ac^	4.17 ± 1.2^Aa^
L	2.5 ± 0.4^Cb^	2.75 ± 0.9^Ab^	2.70 ± 0.6^Ab^	3.43 ± 1.1^Aa^
S	2.42 ± 0.7^Cc^	3.06 ± 1.5^Ab^	2.38 ± 1.6^Ac^	3.76 ± 1.1^Aa^
Volatile fatty acid (ml 0.01 N NaOH/100 ml)				
C	3.22 ± 0.5^Aa^	3.32 ± 0.3^Aa^	2.85 ± 1.2^Ab^	2.35 ± 0.5^Bc^
M	3.83 ± 1.1^Aa^	3.72 ± 1.4^Aa^	2.02 ± 0.2^Ab^	1.93 ± 0.4^Bb^
G	3.12 ± 1.1^Aa^	3.88 ± 1.8^Aa^	2.05 ± 0.2^Ab^	3.10 ± 0.5^Aa^
L	3.20 ± 0.4^Ab^	3.60 ± 0.2^Aa^	2.55 ± 0.2^Ad^	2.97 ± 0.8^Ac^
S	3.37 ± 0.4^Ab^	4.13 ± 1.5^Aa^	2.27 ± 0.0^Ac^	3.13 ± 0.2^Ab^

C: control yoghurt M: yoghurt containing *Gundelia tournefortii* L. milk, G: yoghurt containing *Gundelia tournefortii* L. gum, L: yoghurt containing *Gundelia tournefortii* L. leave, S: yoghurt containing *Gundelia tournefortii* L. stem

^*^ Presented values are the means (± SD) of three replicate trials

^a–^^d^Means in the same raw with different letters were significantly different at *p* < 0.05

^A^^−^^C^Means that, in the same column, different letters were significantly different at *p* < 0.05

The acetaldehyde content of the control yoghurt sample showed the highest value on the first day of storage, followed by the samples M, G, L and S, respectively. There was a statistical difference between the yoghurt samples only on the first day of storage (*p* < 0.05). The acetaldehyde content of yoghurt samples containing *Gundelia tournefortii* L. increased during storage, a kind of change which was found to be statistically significant (*p* < 0.05). Similar results were also reported in other yoghurt samples containing *Moringa oleifera* leaf powder (Hassan et al. 2016). It seemed that yoghurt samples with high acidity contained high levels of acetaldehyde, a result which is consistent with the titration acidity and lactic acid bacteria values observed in the study. Acetaldehyde is formed as a result of glucose metabolism as well as the reaction of the amino acid threonine with the enzyme threonine aldolase and then methionine reaction. In addition, acetaldehyde continues to form in yoghurt during cooling and storage. Therefore, it is thought that the amount of acetaldehyde increases during the storage process due to the high protein content of yoghurts prepared with the addition of *Gundelia tournefortii* L. is added.

The values of the volatile fatty acids of the yoghurt samples—G, L and S—were found to be higher on the 21st day of storage than those found in the M and control samples (*p* < 0.05). Hassan et al. (2016) found that the volatile fatty acid values in the yoghurt samples to which they added *Moringa oleifera* were higher than those in the control yoghurt sample and that such rates increased during storage. The aforementioned researchers explained that this may be due to the composition of the moringa, as well as the contribution of amino acids formed as a result of proteolysis to the formation of some volatile fatty acids. In this study, a similar situation was observed in yoghurt samples (L, S) with added *Gundelia tournefortii* L. parts. While the changes in the amount of volatile fatty acids of yoghurt samples, namely L and S, were statistically significant during storage, they showed a decline after the 7th day in M, G and C yoghurt samples (*p* < 0.05).

### Rheological, structural and colour characteristics of the yoghurt samples

The gel firmness, whey separation, water holding capacity, viscosity and colour of yoghurt samples during storage are presented in Table 3. When compared to those in the control yoghurt sample, gel firmness values were higher in the samples with *Gundelia tournefortii* L. on the 7th and 14th days of storage, in addition to the yoghurt samples in which *Gundelia tournefortii* L. was added, except for the sample L, on the 21st day. This difference was statistically significant only on the 7th and 14th days of storage (*p* < 0.05). There was a decrease in the gel firmness values of all yoghurt samples during storage, which was statistically significant in samples C, G and L (*p* < 0.05). The difference in the gel firmness values of yoghurts may be due to the different composition of the parts of the plant with high protein content. The gel firmness value of yoghurt decreases as a result of the rearrangement of casein bonds, which are in a dynamic equilibrium at the beginning of storage, depending on time and acidity development.

**Table 3:** Rheological, structural and color characteristics of yoghurt samples during storage^*^

	Storage days			
	1	7	14	21
Gel firmness (1/10 mm)				
C	216 ± 3.9^Aa^	197 ± 8^Cab^	196 ± 22^Aab^	184 ± 17^Bb^
M	210 ± 2.8^Aa^	225 ± 9^Aa^	208 ± 15^Aa^	203 ± 16^Aa^
G	219 ± 6.1^Aa^	209 ± 4^Bca^	201 ± 18^Aa^	194 ± 15^ABa^
L	224 ± 15^Aa^	216 ± 5^ABb^	208 ± 12^Ab^	175 ± 12^Bc^
S	216 ± 12^Aa^	205 ± 5^BCa^	213 ± 9^Aa^	205 ± 14^Aa^
Whey separation (%)				
C	29.96 ± 1.7^Aa^	25.79 ± 2.9^Aa^	28.68 ± 2.5^Aa^	28.78 ± 4.6^Aa^
M	30.32 ± 2.9^Aa^	28.29 ± 5.4^Aa^	27.93 ± 4.6^Aa^	28.90 ± 4.2^Aa^
G	31.19 ± 1.5^Aa^	26.65 ± 4.8^Ac^	29.24 ± 2.6^Ab^	29.27 ± 3.8^Ab^
L	29.98 ± 2.1^Aa^	26.72 ± 3.9^Aa^	27.17 ± 4.4^Aa^	28.51 ± 3.8^Aa^
S	28.05 ± 1.5^Aa^	28.18 ± 2.5^Aa^	25.83 ± 3.4^Aa^	27.65 ± 1.9^Aa^
Water holding capacity (%)				
C	51.47 ± 4.9^Aa^	46.03 ± 2.6^Ab^	45.17 ± 5.1^Ab^	45.00 ± 3.5^Ab^
M	50.77 ± 3.3^Aa^	46.97 ± 3.3^Ab^	45.47 ± 2.3^Ab^	45.20 ± 1.7^Ab^
G	51.37 ± 2^Aa^	48.67 ± 4.1^Ab^	48.40 ± 3.6^Ab^	46.23 ± 4.2^Ab^
L	51.80 ± 3.2^Aa^	47.00 ± 6.9^Ab^	44.47 ± 5.1^Ab^	46.17 ± 0.9^Ab^
S	50.20 ± 1.8^Aa^	46.83 ± 5.8^Ab^	43.47 ± 4.8^Ac^	43.80 ± 3^Ac^
Viscosity (cP)				
C	1486 ± 92^Aab^	1670 ± 51^Aa^	1095 ± 268^Ab^	1930 ± 74^Aa^
M	1273 ± 85^Ba^	1382 ± 108^Ba^	1524 ± 234^Aa^	1656 ± 272^ABa^
G	1146 ± 37^Bb^	1252 ± 99^ABb^	1420 ± 293^Aa^	1565 ± 195^ABa^
L	1148 ± 46^Bb^	1130 ± 99^Cb^	1401 ± 215^Aa^	1375 ± 96^Ba^
S	1202 ± 62^Bd^	1358 ± 210^ABc^	1492 ± 240^Ab^	1658 ± 240^ABa^
L*				
C	94.59 ± 1.1^Aa^	94.20 ± 0.6^Aa^	90.53 ± 7.1^Aa^	94.52 ± 0.9^Aa^
M	94.46 ± 0.3^Aa^	91.80 ± 2.8^Aa^	93.94 ± 0.1^Aa^	94.30 ± 0.5^Aa^
G	94.28 ± 0.3^Aa^	92.43 ± 2.9^Aa^	91.55 ± 4.6^Aa^	93.78 ± 0.7^Aa^
L	93.98 ± 1.2^Aa^	92.89 ± 3.4^Aa^	94.11 ± 0.8^Aa^	93.92 ± 1.1^Aa^
S	94.38 ± 0.2^Aa^	93.06 ± 2.8^Aa^	93.39 ± 0.8^Aa^	93.63 ± 0.8^Aa^
a*				
C	− 4.19 ± 0.6^Aa^	− 4.18 ± 0.3^Aa^	− 4.05 ± 0.6^Aa^	− 4.03 ± 0.5^Aa^
M	− 4.20 ± 0.5^Aa^	− 4.41 ± 0.1^Aa^	− 3.68 ± 1.1^Aa^	− 3.41 ± 1.1^Aa^
G	− 4.13 ± 0.5^Aa^	− 3.91 ± 0.5^Aa^	− 3.64 ± 0.7^Aa^	− 3.47 ± 0.6^Aa^
L	− 4.13 ± 0.6^Ac^	− 3.95 ± 0.4^Ab^	− 3.58 ± 0.8^Aa^	− 3.61 ± 0.6^Aa^
S	− 4.07 ± 0.4^Ac^	− 3.89 ± 0.3^Ab^	− 3.63 ± 0.9^Ab^	− 3.25 ± 0.4^Aa^
b*				
C	13.55 ± 1.3^Ab^	13.35 ± 1.1^Ab^	16.48 ± 3.94^Aa^	13.18 ± 1.6^Ab^
M	13.44 ± 1.4^Aa^	14.05 ± 0.7^Aa^	13.52 ± 0.80^Aa^	12.77 ± 0.2^Aa^
G	13.55 ± 1.9^Ab^	14.55 ± 0.7^Aab^	16.99 ± 4.95^Aa^	12.87 ± 3.6^Ac^
L	13.41 ± 1.6^Aa^	12.79 ± 1.8^Ab^	12.44 ± 1.63^Ab^	13.30 ± 0.2^Aa^
S	13.13 ± 1.3^Aa^	13.10 ± 1.4^Aa^	13.11 ± 1.50^Aa^	13.78 ± 1.3^Aa^

C: control yoghurt M: yoghurt containing *Gundelia tournefortii* L. milk, G: yoghurt containing *Gundelia tournefortii* L. gum, L: yoghurt containing *Gundelia tournefortii* L. leave, S: yoghurt containing *Gundelia tournefortii* L. stem

^*^ Presented values are the means (± SD) of three replicate trials

^a–^^d^Means in the same raw with different letters were significantly different at *p* < 0.05

^A^^−^^C^Means that in the same column, different letters were significantly different at *p* < 0.05

Whey separation values appeared to be close to each other in all yoghurt samples on all days of storage (*p* > 0.05). The whey separation values of the yoghurt samples in the study were found to be lower than those reported as 18–71.85% for the yoghurt samples with chickpea extract (Aguilar-Raymundo and Velez-Ruiz 2019) and 36.11% for that containing chickpea (Güzeler et al. 2019). Whey separation values of yoghurt samples decreased during storage, but such a decrease was statistically significant only in sample G until day 14 (*p* < 0.05). The decreased whey separation during storage may be associated with the increased titratable acidity values of yoghurt samples during storage and with their being stored at a low temperature. Moreover, Ebrahim et al. (2015) reported that whey separation values in the yoghurt samples enriched with *Gundelia tournefortii* decreased during storage, a result which is in conformity with that of the present study.

In the yoghurt samples in which *Gundelia tournefortii* L. was added, the highest water holding capacity (WHC) was obtained in yoghurt sample G with a percentage of 46.23 in 21 days of storage. No statistical difference was observed between the WHC values of the control sample and other yoghurt samples (*p* > 0.05). In this context, Güzeler et al. (2019) reported that the water holding capacity of a chickpea yoghurt sample was 39.50%. The WHC of yoghurt samples decreased during storage. Similar result has been reported by El-Nawasany 2019. Such a decline during storage was statistically significant only until day 15 in the sample S and up to day 7 in all other samples (*p* < 0.05). The high acidity and casein aggregation from lactic acid fermentation promote large and weaking of the curd which eventually leads to noticeable whey separation and a decrease in the water holding capacity in yoghurt. Moreover, yoghurt with fruits, vegetables and plant due to content of dry matter and pectin increases the water holding capacity.

While it was the control sample that had the highest viscosity value on the 1st, 7th and 21st days of storage, the yoghurt sample M appeared to have the highest value on the 14th day of storage. The difference between the viscosity values of the control sample and the yoghurt sample to which *Gundelia tournefortii* L. was added was statistically significant except for the case on day 14 (*p* < 0.05). Hassan et al. (2016) reported that the viscosity of the control sample was higher than the yoghurt sample containing *Moringa oleifera* leaf powder and the viscosity value increased in all samples during storage. During the storage period, there were increased viscosity values in the yoghurt samples, a result which was statistically significant in the other yoghurt samples except for the sample M (*p* < 0.05). The increased viscosity values of yoghurt samples during storage can be explained by the bacterial activity that decreases the pH and increases the strength of the protein network (Ebrahimi and Sani 2015). In this connection, El-Nawasany (2019) reported results of their study on a stirred yoghurt sample containing Vitex agnus-castus; the results were similar to those of this study.

Colour is one of the most important visual features in yoghurt and is an important parameter in consumer preference. The parameter L* of the yoghurt samples changed between 90.53 and 94.59 during storage and showed high brightness values. While parameter a* changed between − 3.25 and − 4.41 with the tendency to green, parameter b* took values between 12.44 and 16.99 with a tendency to yellow. However, statistical analysis showed that there was no significant difference (*p* > 0.05) between L*, a* and b* values of all yoghurt samples. A slight decrease in L* values was observed during the 21-day storage period of yoghurt samples, although not statistically significant (*p* > 0.05). Such results are consistent with those of Hassan et al. (2016), who reported that the L value decreased during storage. In all samples, a* value maintained negative values (green colour range) during storage, whereas a* values appeared to have increased in all samples at the end of storage when compared to those values obtained on the first day. Except for the samples L and S, the changes in a* values of yoghurt samples during storage did not seem to be statistically significant (*p* > 0.05). The b* values of yoghurt samples decreased at the end of storage, which was found to be statistically significant in the samples C, G and L (*p* < 0.05).

### Microorganism counts in the yoghurt samples

**Fig. 1:**
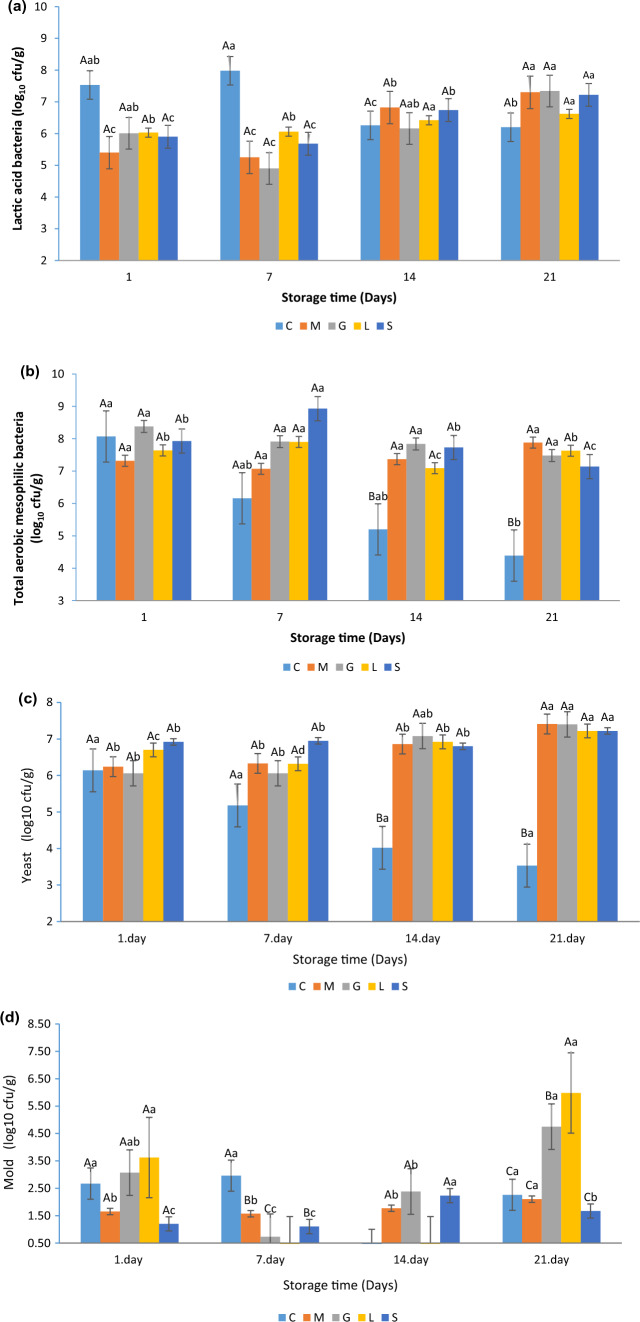
Changes in counts of lactic acid bacteria **a**, total mesophilic aerobic bacteria **b**, yeast **c** and mold **d** in yoghurts during storage. C: Control yoghurt M: yoghurt containing *Gundelia tournefortii* L. milk, G: yoghurt containing Gundelia tournefortii L. gum, L: yoghurt containing Gundelia tournefortii L. leave, S: yoghurt containing Gundelia tournefortii L. stem.^a–d^Chart bars with different letters were significantly different at *p* < 0.05 for storage.^A−C^Chart bars with different letters were significantly different at *p* < 0.05 for between yoghurt samples

Changes in the counts of lactic acid bacteria, total aerobic mesophilic bacteria, yeast and mould in yoghurt samples during storage are shown in Fig. 1. There was no statistical difference in terms of lactic acid bacteria (LAB) between the control yoghurt sample and those containing *Gundelia tournefortii* L. (*p* > 0.05). Güzeler et al. (2019) reported the number of lactic acid bacteria in the yoghurt sample containing chickpeas as 4.72 log CFU/g, which was lower than the values determined in the present study. While the number of LAB decreased in the control sample of yoghurt during storage, it increased in the samples produced by adding *Gundelia tournefortii* L. These changes during storage were statistically significant for all samples (*p* < 0.05). This difference was found to be statistically significant for the samples C and G during storage, while it was significant for the samples M, L and S after the 15th day of storage. It is desirable that the bacteria survive in large numbers in yoghurt (at least, 10^6^ CFU/g) until the expiration day (Wajs et al. 2023). The bacterial count of the yoghurt samples in the study increased to the extent that it became > 6 log CFU/g at the end of storage process. The use of various plants in yoghurt formulation tends to increase the activity and viability of Streptococcus and Lactobacillus bacteria during storage. This increased vitality can be attributed to the polyphenols and fibres contained in the plant-derived food (Dhawi et al. 2020).

The highest total number of aerobic mesophilic bacteria (TAMB) was found in the yoghurt sample S on day 7 of storage, whereas the lowest in control yoghurt on day 21 of storage. The difference between the TAMB in the control sample and the yoghurt samples containing *Gundelia tournefortii* L. was statistically significant on the 14th and 21st days (*p* < 0.05). During storage, the TAMB decreased in the other yoghurt samples except the yoghurt sample M, and the control sample had the lowest TAMB count. The decrease in the TAMB was found to be statistically significant only in the samples C, L and S during storage (*p* < 0.05). El-Nawasany (2019) observed that the total bacterial count of yoghurt samples containing different ratios of vitex agnus-castus increased gradually during storage, reaching the highest number at the end of storage.

Yeast counts of yoghurt samples containing *Gundelia tournefortii* L. ranged from 6.06 log CFU/g to 7.41 log CFU/g (*p* > 0.05). On the 14th and 21st days of storage, the yeast counts of the control sample turned out to be lower than those of the yoghurt samples containing *Gundelia tournefortii* L., and the difference between them was found to be statistically significant (*p* < 0.05). According to recent study, it can be suggested that the fruit and plant added to yoghurt are effective on yeast and mold growth. Yeast counts of the yoghurt containing *Gundelia tournefortii* L. increased during storage, a result which was found to be statistically significant (*p* < 0.05). Some researchers have similarly reported that the yeast and mold counts of yoghurt samples containing vitex agnus-castus increased during 15-day storage (El-Nawasany et al. 2019).

There was no significant difference between the mold counts of the yoghurt samples on the first day of storage (*p* > 0.05). On the 7th and 14th days, no mold appeared in the sample L, while the sample S had the lowest mould count on the 21st day. In another study, yeast and mold growth were not detected on the first day of 14-day storage in yoghurt samples containing rosemary extracts. However, after the 7th day, they reported that the yeast and mold counts of yoghurts were 1.30–1.33 CFU/g (Ali et al. 2021). A similar result was obtained in our study, indicating that the storage period affected the growth and activity of mold (*p* < 0.05). The higher than expected yeast and mold values in the yoghurt samples can be explained by the added *Gundelia tournefortii* L. It can also be explained by the presence of contamination in the analysed samples and storage conditions. Microbiological analysis showed that the yoghurt samples containing *Gundelia tournefortii* L. had the highest counts of lactic acid bacteria at the end of the cold storage.

### Sensory properties of the yoghurt samples

**Fig. 2:**
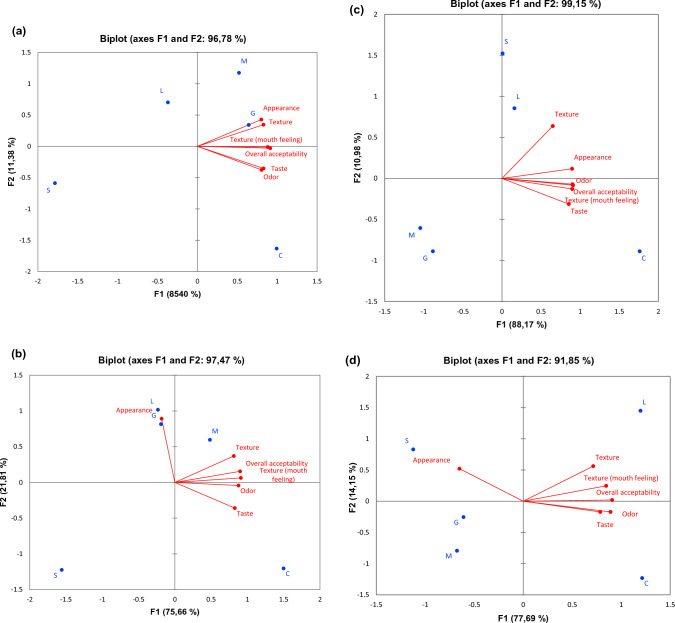
Principal component analysis of sensory properties of yoghurt samples **a** day1, **b** day7, **c** day14, **d** day21. C: control yoghurt M: yoghurt containing *Gundelia tournefortii* L. milk, G: yoghurt containing *Gundelia tournefortii L.* gum, L: yoghurt containing *Gundelia tournefortii L.* leave, S: yoghurt containing *Gundelia tournefortii L.* stem

The results of the PCA of sensory attributes of yoghurt samples are provided in Fig. 2, showing that the PCA biplot of yoghurt samples indicates certain differences from one sample to another. The percentage changes for the first and second components were 96.78%, 97.47%, 99.15% and 91.85% for the 1st, 7th, 14th, and 21st days respectively, during which the yoghurt samples were stored. The samples were divided into four groups on the score chart during storage. Until the 7th day of storage, the sample M was on the upper right, while yoghurt sample L was on the upper left. The sample G was placed on the left side after day 1.

**Fig. 3:**
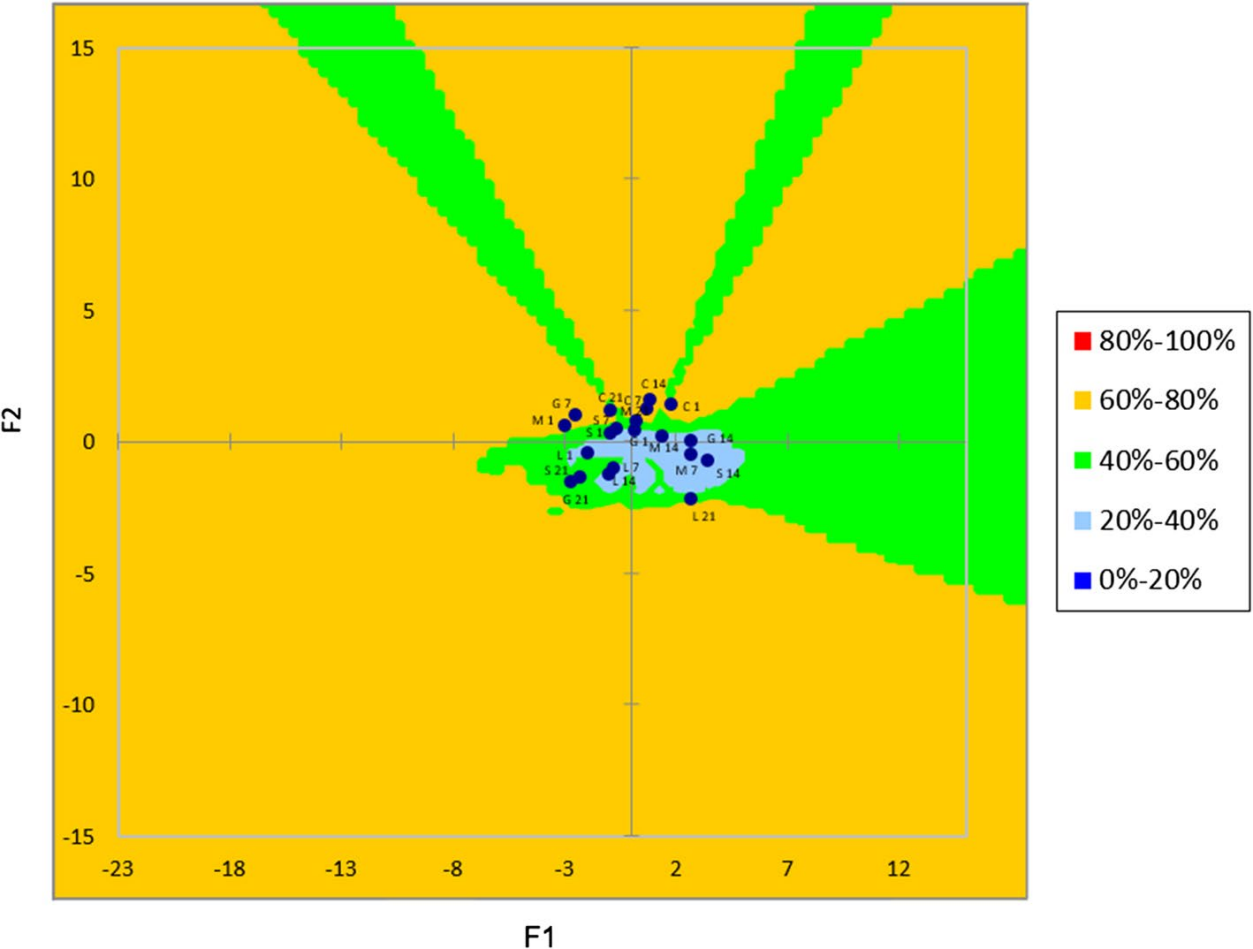
External preference map for yoghurts. C: control yoghurt M: yoghurt containing *Gundelia tournefortii* L. milk, G: yoghurt containing *Gundelia tournefortii L.* gum, L: yoghurt containing *Gundelia tournefortii L.* leave, S: yoghurt containing *Gundelia tournefortii L.* stem

After the PCA was conducted on the yoghurt samples grouped according to the panellists’ preferences by performing agglomerative hierarchical clustering (AHC), an external preference map was created as shown in Fig. 3. Except for the first day of storage, all control yoghurt samples (C7, C14, and C21) and sample G on the 7th day of storage were located in the orange region, indicating that the yoghurt samples were preferred by the 60–80% of all the panellists. The yoghurt samples M, G and S were in the preference range of 40–60% on the 21st day of storage, whereas yoghurt C was there on the 1st day of storage. The yoghurt sample L stored for 21 days and the M at the beginning of the storage were at the border of these two regions. In contrast, the yoghurt sample G included in the light blue area seems to have satisfied only a small percentage of consumers (20–40%) in total on days 1 and 14 of storage, just like the sample M on days 7 and 14, the sample L on days 1, 7 and 14, and sample S on the day 14 of storage. The sample S remained at the border between the light-blue and green zone on the 1st and 7th days of storage.

It was observed that the acidity in yoghurt containing *Gundelia Tournefortii* L. increased gradually due to bacterial activity, but it did not significantly affect sensory properties such as odour, taste and texture during storage. However, it was seen that the appearance scores of yoghurts decreased.

## Conclusion

Yoghurt samples were produced by adding *Gundelia tournefortii* L. milk, gum, leaves and stems and stored at 4 °C for 21 days. Of all the yoghurt samples, the highest development of a macro element was detected in the sample M, and the highest Zn content was seen in the sample G. Acetaldehyde and gel firmness values of yoghurt samples containing *Gundelia tournefortii* L. had higher values compared to those of the control sample. Likewise, pH, whey separation and WHC values were found to be similar to those of the control sample. While no difference was observed between the L*, a* and b* values of yoghurt samples, it also appeared that L*and b* values decreased, though a* increased in value during storage. The number of lactic acid bacteria was found to be greater than 10^6^ CFU/g. PCA of sensory properties shows that yoghurt samples were distributed into four groups on different days of storage. The PCA has also revealed that the control yoghurt sample had higher overall acceptability value compared to those with added *Gundelia tournefortii* L, but that only the samples M and G presented acceptable scores at the beginning of storage, with the sample L doing so at the end of storage. Based on all these results, it was concluded that the samples M, G and L are acceptable in terms of their high nutritional value and of their physicochemical, microbiological and sensory properties, signifying that they can be offered to consumers as alternative yoghurt. Further research should be carried out to determine the health-related properties of yoghurt with animal models and human clinical trials. Therefore, this study may provide base information for future studies.

## Supplementary Information

Below is the link to the electronic supplementary material.

Supplementary file1 (DOC 50 KB)

Supplementary file2 (DOC 39 KB)

Supplementary file3 (DOC 50 KB)

## Data Availability

Not applicable.
