# Supplementary material for: Physicochemical, colour, microbiology, sensory and mineral attributes of set-type yoghurt produced from Gundelia tournefortii L. and its gum
Source: J Food Sci Technol. 2024 May 3;61(11):2166–76. doi: 10.1007/s13197-024-05987-1 (PMC11464860; doi:10.1007/s13197-024-05987-1)
Supplement: Supplementary file 1 — Supplementary file1 (DOC 50 KB) [file 13197_2024_5987_MOESM1_ESM.doc]

**Table S1** Dry matter, fat and protein contents of yoghurts samples^*^

| Samples | Dry matter (%) | Fat (%) | Protein (%) |
| --- | --- | --- | --- |
| C | 13.22±0.4^a^ | 3.1±0.4^a^ | 4.11±0.9^a^ |
| M | 13.32±0.5^a^ | 3.0±0.4^a^ | 4.46±1.1^a^ |
| G | 13.38±0.8^a^ | 3.0±0.4^a^ | 4.63±1.3^a^ |
| L | 13.14±0.6^a^ | 2.96±0.4^a^ | 5.11±1.6^a^ |
| S | 12.95±0.5^a^ | 3.1±0.2^a^ | 5.0±0.7^a^ |

C: Control yoghurt M: yoghurt containing *Gundelia tournefortii* L. milk, G: yoghurt containing Gundelia tournefortii L. gum, L: yoghurt containing Gundelia tournefortii L. leave, S: yoghurt containing Gundelia tournefortii L. stem

* Presented values are the means (±SD) of three replicate trials.

^a,b,c,d^ Means that, in the same column, different letters were significantly different at p < 0.05.
