# Supplementary material for: Physicochemical, colour, microbiology, sensory and mineral attributes of set-type yoghurt produced from Gundelia tournefortii L. and its gum
Source: J Food Sci Technol. 2024 May 3;61(11):2166–76. doi: 10.1007/s13197-024-05987-1 (PMC11464860; doi:10.1007/s13197-024-05987-1)
Supplement: Supplementary file 2 — Supplementary file2 (DOC 39 KB) [file 13197_2024_5987_MOESM2_ESM.doc]

12.10.2023

**Dear Editor in Chief,**

Enclosed please find my original research manuscript entitled "**Physicochemical, colour, microbiology, sensory and mineral attributes of set-type yoghurt produced from *Gundelia tournefortii* L. and its gum**" which I am submitting for your consideration as a publication in Journal of Food Science and Technology.

With the increasing importance of health, it has now become possible to develop alternative dairy products by including herbal ingredients with rich nutritional content and functional properties and it was lead to an innovative concept in the food science and technology. This situation also revealed the necessity of conducting new researches. In this study, therefore, a yoghurt sample was produced by using the parts of *Gundelia tournefortii* L. (L, S), its milk (M) and the gum (G) obtained from the milk, in yoghurt so as to compare their properties. Yoghurt samples were stored at refrigerator temperature and their physicochemical, microbiological and sensory properties were identified on the 1st, 7th, 14th, and 21st days. Furthermore, the mineral contents of the yoghurt samples were established in this study, in which their Ca, P, K, Na and Mg contents were found to be higher than those of the control sample. Of all the yoghurt samples, the highest development of a macro element was detected in the sample M, and the highest Zn content was seen in the sample G. It was also determined that the acetaldehyde and gel firmness values of the yoghurt samples were higher than those of the control yoghurt, and that the lactic acid bacteria count on the 21st day was > 6 log CFU/g in all yoghurt samples. The preference map demonstrated that the yoghurt samples were placed in different percentages. Among these yoghurts, the samples M, G and L were found to have acceptable scores. According to these research results, parts and gums of *Gundelia tournefortii* L. turned out to be suitable for use in yoghurt production.

The total word counts of the manuscript are as follows: MS: 5607, Abstract: 190, References: 30, Tables and Figures: 3 and 3, respectively.

The highlights of the work are presented below;

- *Gundelia tournefortii* L. parts were used for yoghurt production.
- The Gundelia tournefortii L. yoghurt was high in Ca, P, K, Na, Mg.
- The lactic acid bacteria count of yoghurt samples increased at the end of storage.
- Adding *Gundelia tournefortii* L. into the yoghurt was ensured acceptable sensory scores.

The data and information contained in this manuscript have not been published elsewhere nor are they included in any manuscript currently under review by another journal. Author of this manuscript has any financial or other kind of conflict of interest related to this work. Be assured that author of this paper did contribute significantly to the conception, design, execution, data analysis and interpretation or writing associated with this study and report.

I reviewed two manuscripts in 2018, two manuscripts in 2019, one manuscript in 2020, five manuscript in 2021 for Journal of Food Science and Technology and I shall review at least three manuscripts (in my own specialization) submitted to JFST.

Thank you for considering this paper.

Sincerely,

**Assoc. Prof. Dilek SAY**

Vocational School of Pozantı

University of Cukurova

01470 Pozantı / Adana

TURKEY

Mobile: +90 532 307 97 16

e-mail: [dsay@cu.edu.tr](mailto:dsay@cu.edu.tr) or dileksay@gmail.com

ORCID ID:0000-0003-3932-6414
